# Excitation-secretion coupling in chromaffin cells of the adrenal gland: Where does calcium come from?

**DOI:** 10.1007/s00424-023-02867-z

**Published:** 2023-10-16

**Authors:** Petronel Tuluc, Emilio Carbone

**Affiliations:** 1 Department of Pharmacology and Toxicology, Center for Molecular Biosciences, University of Innsbruck, A-6020 Innsbruck, Austria; 2 Department of Drug Science, NIS Centre, University of Torino, IT-10125 Turin, Italy

Following the sympathetic nervous system activation, the chromaffin cells (CCs) of the adrenal medulla respond with a membrane depolarization and trains of action potentials (APs) that activate the high voltage gated Ca^2+^ channels (HVCCs). The HVCC Ca^2+^ influx triggers the fusion of catecholamine (CA)-containing vesicles with the plasma membrane and CAs are released in the circulation. From the very first studies, it has been clear that stimulus-secretion coupling in CCs is a Ca^2+^-dependent process and HVCCs activation and Ca^2+^ influx are fundamental [1, 6, 11]. However, this does not imply that HVCC Ca^2+^ influx is sufficient to sustain the whole CA secretion. Indeed, it has been recently shown that CA release depends on the concerted contribution of HVCC Ca^2+^ influx, Ca^2+^-induced Ca^2+^-release (CICR) from intracellular Ca^2+^ stores and mitochondrial Ca^2+^ buffering [4, 5]. The contribution of the different Ca^2+^ sources seems to be species specific as acute reversible SERCA pump inhibition using cyclopizonic acid (CPA) reduces CA release in bovine CCs but enhances it in mouse CCs [8].

In the current issue of the Pfuggers Archive, *Oscar J. Parada-Parra* and *Arturo Hernández-Cruz* extended this analysis to cultured CCs from normotensive (WKY) and spontaneously hypertensive (SHR) rats [10]. Previously, it has been shown the SHR CCs can elicit a stronger CA release compared to WKY CCs primarily due to a stronger CICR mechanism. Despite these differences, the current study demonstrates that SERCA pump inhibition using CPA equally suppresses CA release in both WKY and SHR CCs. As shown by the authors, CPA application leads to an almost complete reduction in vesicle exocytosis that correlates with the dramatic decrease in intracellular Ca^2+^ transients. This unequivocally demonstrates that in rat CCs Ca^2+^ release from intracellular stores represents the major determinant of the excitation-secretion coupling mechanism. Therefore, a model emerges where plasma membrane HVCC Ca^2+^ influx is required to activate the RyRs and intracellular store release while the global increase in Ca^2+^ concentration is what controls CA vesicle exocytosis (Fig. 1). This seems to be remarkably similar to the cardiac excitation-contraction coupling mechanism where Cav1.2 channel activation leads to a local increase in Ca^2+^ concentration that in turn activates the intracellular RyRs and trigger Ca^2+^ release from sarcoplasmic reticulum [2].

**Fig. 1:**
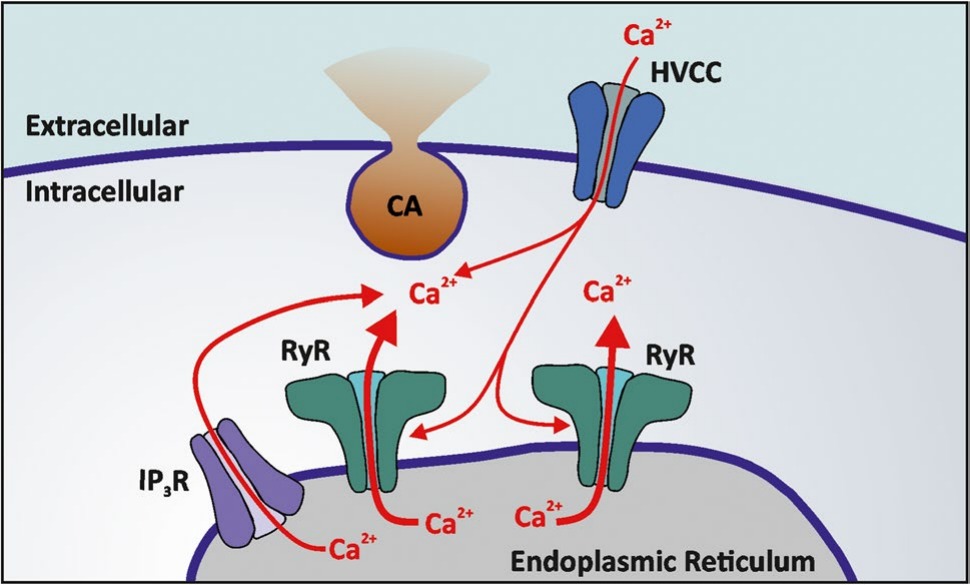
Ca^2+^ sources contributing to CA exocytosis in rat CCs. HVCC Ca^2+^ influx acts primarily to activate the RyRs localised in endoplasmic reticulum (ER) membrane. ER Ca^2+^ release together with HVCC Ca^2+^ influx trigger CA vesicle fusion with the plasma membrane and hormone release

While the study provides exciting novel findings regarding CCs signaling, it also opens a number of questions. Exocytosis was measured using amperometry in response to 1-s-long KCl-induced membrane depolarization which allows for a prolonged summation of the Ca^2+^ signal. Given the existence of different neuronal firing modes of CCs [7], it would be interesting to know what would be the contribution of the store release during repetitive firing and rhythmic bursting of APs. CCs express several HVCC isoforms [3, 7]. Thus, a second reasonable question could be which HVCC isoform is preferentially coupled to store release and vesicle exocytosis? Perhaps the most interesting finding of the manuscript is that rat CCs show a large functional variability with different contributions of the HVCC Ca^2+^ influx and ER-store release to intracellular Ca^2+^ transients and vesicle exocytosis. Are these differences existing also in CCs from adrenal slices or in vivo? Is the contribution of ER release identical in health and disease? What cellular mechanisms control this variability?

Because of their very robust exocytosis and ease of CA detection using amperometry [12], CCs were the first model cell system where neurotransmitter release has been investigated [1]. Over the decades, CCs research contributed with crucial information regarding the excitation-secretion coupling mechanism [3] and still remains a valid cell model for studying the complexity of the highly regulated process of neurosecretion [9]. Nevertheless, the study from *Oscar J. Parada-Parra* and *Arturo Hernández-Cruz* [10] demonstrates that many questions are still unanswered and CCs research can still uncover key information on the physiology and pathophysiology of Ca^2+^-dependent neurotransmission.

## Data Availability

Not applicable.
